# Synthesis and Antiplasmodial Activity of Novel Bioinspired Imidazolidinedione Derivatives

**DOI:** 10.3390/biom11010033

**Published:** 2020-12-29

**Authors:** Anna Jaromin, Anna Czopek, Silvia Parapini, Nicoletta Basilico, Ernest Misiak, Jerzy Gubernator, Agnieszka Zagórska

**Affiliations:** 1 Department of Lipids and Liposomes, Faculty of Biotechnology, University of Wroclaw, 50-383 Wroclaw, Poland; jerzy.gubernator@uwr.edu.pl; 2 Department of Medicinal Chemistry, Faculty of Pharmacy, Jagiellonian University Medical College, Medyczna 9 str, 30-688 Kraków, Poland; anna.czopek@uj.edu.pl (A.C.); ernest.misiak@student.uj.edu.pl (E.M.); agnieszka.zagorska@uj.edu.pl (A.Z.); 3 Dipartimento di Scienze Biomediche per la Salute, Università di Milano, Via Pascal 36, 20133 Milan, Italy; silvia.parapini@unimi.it; 4 Dipartimento di Scienze Biomediche, Chirurgiche e Odontoiatriche, Università di Milano, Via Pascal 36, 20133 Milan, Italy; nicoletta.basilico@unimi.it

**Keywords:** Imidazolidine-2,4-dione derivatives, *Plasmodium falciparum*, antiparasitic agents, antimalarial, drug resistance, cytotoxicity, hemolysis

## Abstract

Malaria is an enormous threat to public health, due to the emergence of *Plasmodium falciparum* resistance to widely-used antimalarials, such as chloroquine (CQ). Current antimalarial drugs are aromatic heterocyclic derivatives, most often containing a basic component with an added alkyl chain in their chemical structure. While these drugs are effective, they have many side effects. This paper presents the synthesis and preliminary physicochemical characterisation of novel bioinspired imidazolidinedione derivatives, where the imidazolidinedione core was linked via the alkylene chain and the basic piperazine component to the bicyclic system. These compounds were tested against the asexual stages of two strains of *P. falciparum*—the chloroquine-sensitive (D10) and chloroquine-resistant (W2) strains. In parallel, in vitro cytotoxicity was investigated on a human keratinocyte cell line, as well as their hemolytic activity. The results demonstrated that the antiplasmodial effects were stronger against the W2 strain (IC_50_ between 2424.15–5648.07 ng/mL (4.98–11.95 µM)), compared to the D10 strain (6202.00–9659.70 ng/mL (12.75–19.85 µM)). These molecules were also non-hemolytic to human erythrocytes at a concentration active towards the parasite, but with low toxicity to mammalian cell line. The synthetized derivatives, possessing enhanced antimalarial activity against the CQ-resistant strain of *P. falciparum*, appear to be interesting antimalarial drug candidates.

## 1. Introduction

Malaria is a life-threatening disease caused by parasites that are transmitted to people through the bites of infected female *Anopheles* mosquitoes. Malarial parasites belong to the *Plasmodium* genus, which include different species, with two of these species posing the greatest threat, namely, *Plasmodium falciparum* and *Plasmodium vivax*. According to the World Health Organization (WHO), in 2018, *P. falciparum* accounted for 99.7% of the estimated malaria cases in the African Region, 50% of cases in the South-East Asia Region, 71% of cases in the Eastern Mediterranean and 65% in the Western Pacific [1]. *P. vivax* is the predominant parasite in the WHO Region of the Americas, representing 75% of malaria cases. Moreover, in 2018, nearly half of the world’s population was at risk of malaria, and malaria remains an important cause of illness and death in children and adults in countries in which it is endemic.

A malaria infection is characterized by a spectrum of signs and symptoms such as fever, chills, headache, nausea (vomiting), muscle pain, sweating and cough with specific cycles of “attacks”. An attack usually starts with shivering and chills, progressing to a high fever, followed by sweating and a return to normal temperature. Symptoms of malaria are caused by the asexual cycle of *Plasmodium* parasites in the blood of the human host. When an infected female *Anopheles* mosquito takes a blood meal, sporozoites are injected into the bloodstream and circulate to infect the liver. During the liver stage, the sporozoites mature into tissue schizonts, containing thousands of merozoites, which are released back into the bloodstream, where they infect red blood cells. During the blood stage, the parasites further develop through ring, trophozoite and blood schizont stages, producing new merozoites that infect more healthy red blood cells. Within the erythrocytes, repeated cycles of parasite replication occur, causing a loss of red blood cells.

A variety of antimalarial drugs have been developed, based on the species of malaria parasite, the severity of the symptoms, age, and general condition of infected people. The most common antimalarial drugs include artemisinin derivatives and blood schizonticidal drugs with a quinoline scaffold. For instance, quinine (Q), chloroquine (CQ), primaquine (PQ), amodiaquine (AQ), and mefloquine (MQ) are fast-acting and highly effective blood schizonticidal drugs that are active against sensitive malaria parasites, primarily *P. falciparum* (Table 1) [2]. In fact, new antimalarial drugs are being researched and developed as a consequence of the constant struggle between evolving drug-resistant parasites and the search for new drug formulations to combat them. For example, some isolates of the malaria parasite have demonstrated resistance to nearly all of the available antimalarial drugs.

CQ and its derivatives are based on a 4-aminoquinoline scaffold, which has been extensively used as the chemical core for the synthesis of novel compounds with antimalarial activity. Modifications of the CQ structure mainly consist of changes to the pentamidine chain and amino alkyl head, although recently there have been attempts to exchange the quinoline ring with another heterocyclic ring based on ring isosterism [3,4]. Thus, a cyclic dicarboxamide derivative of chlorproguanil, compound WR182393 (a 2-guanidinoimidazolidinedione derivative) was found to completely eliminate malarial parasites from the body (Table 1) [5]. Several peptide and amino acid derivatives of primaquine and other 8-aminoquinoline antimalarials have been synthesized to reduce the metabolic oxidative deamination pathway, as well as to reduce toxicity of the parent drug. Moreover, imidazolidin-4-ones, prepared from amino acid derivatives of primaquine, exhibit potent gametocytocidal activity against *P. berghei* (Table 1) [6]. Systematic structure-activity relationship studies undertaken on a hit compound, TDR32750 (ethyl 5-methyl-3-oxo-1,2-dihydropyrrole-4-carboxylate), with the aim of improving antiparasitic activity, revealed that replacement of the pyrrolone core on the imidazolidin-2,4-dione gave a similar level of activity against *P. falciparum* (Table 1) [7]. Aplysinopsin is a tryptophan-derived natural marine product, which is composed of an indole coupled with an imidazolidinone moiety [8]. Aplysinopsin exhibited an antiplasmodial activity against FcM29-Cameroon, a high chloroquine-resistant strain of *P. falciparum* (IC_50_ 430 ng/mL (1.69 μM) [9]. Aplysinopsin-type compounds (aplysinopsins) have been reported in many marine organisms from various geographic locations. A variety of biological activities, ranging from antimicrobial and antimalarial to antitumor and neuromodulatory activities have been found in a group of aplysinopsins [10,11].

The hydantoin (imidazolidine-2,4-dione) core is an important pharmacophore that exhibits many biological properties. Diverse hydantoin derivatives are well-known as anticonvulsive (phenytoin, fosphenytoin), antiarrhythmic (azimilide), antimicrobial agents (nitrofurantoin), skeletal muscle relaxants (dantrolene), and nonsteroidal antiandrogen (nilutamide) drugs. Moreover, hydantoin derivatives (substituted at positions C-5, N-1, and N-3, alkyl/arylidene, spiro, polycyclic, and amino hydantoins) have been further explored with the view of their potential medicinal and pharmaceutical properties [12]. Hydantoins exhibit anticonvulsive [13], antidepressant [14], antiviral and antithrombotic activities [15]. Moreover, some of them possess inhibitory activity against enzymes (human aldose reductase and human leucocyte elastase) [16]. The hydantoin core is also present in herbicides (spirohydantoin, thioxohydantocidin), fungicides (clodantoin), and insecticides [17].

**Figure 1:**
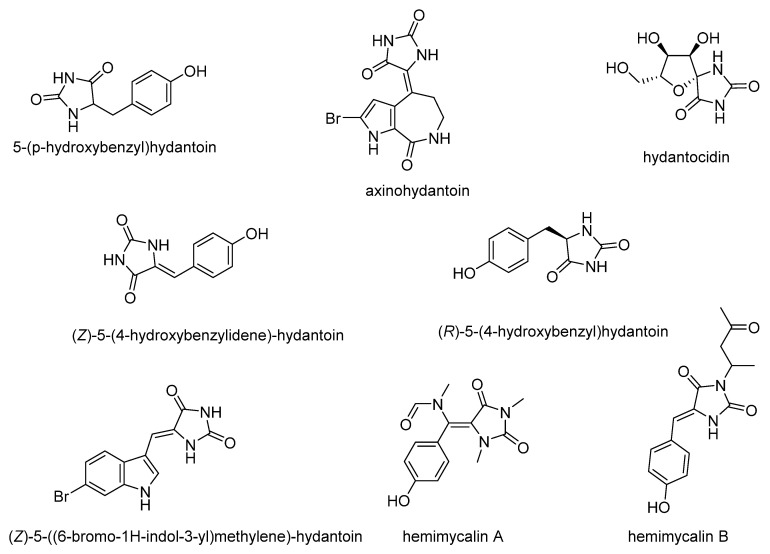
Naturally occurring hydantoins.

Hydantoin and its derivatives are frequently encountered in naturally occurring substances, mostly of marine organisms and bacteria (Figure 1). Endophytic fungus from an estuarine mangrove on the South China Sea coast contain 5-(p-hydroxybenzyl)hydantoin. Many alkaloids have been extracted from sponges or corals, which contain a hydantoin moiety. Most of them exhibited biological activity, such as the well-known aplysinopsins, with cytotoxic properties [8], *Z*-axinohydantoin and debromo-*Z*-axinohydantoin from the Sponge *Stylotella aurantium*, which are potent inhibitors of protein kinase C [18]. Hydantocidin, a spiro nucleoside derived from submerged cultures of *Streptomyces hygroscopicus* SANK 63584 possesses herbicidal and plant growth regulatory activity due to the inhibition of adenylsuccinate synthetase [19]. The ethanolic extract of the abundant shallow water Red Sea sponge, *H. arabica*, contains (*Z*)-5-(4-hydroxybenzylidene)-hydantoin [17], (*R*)-5-(4-hydroxybenzyl)hydantoin and (*Z*)-5-((6-bromo-1*H*-indol-3-yl)methylene)-hydantoin [20], and hemimycalin A i B [17]. Extracts or isolated compounds of this organism inhibited the proliferation and invasion of human prostate cancer PC-3M cell line, exhibited moderate antiproliferative activity against the human cervical carcinoma (HeLa) and displayed variable antimicrobial activities [17,20].

The main purpose of this study is the evaluation of antimalarial activity of new compounds (**5**–**8**) based on the imidazolidine-2,4-dione scaffold presented in naturally occurring hydantoins. Modification of the hydantoin core referred to the various bicyclic moieties attached at the C5 position of hydantoin scaffold by spiro carbon. Next, N3 position of imidazolidine-2,4-dione scaffold were connected via a five-carbon linker with polycyclic- or heteroaryl-piperazine.

Meyers et al. [21] identified spiropiperidine hydantoins as new leads for antimalarial drug discovery. Lead compound CWHM-123 (8-(5-chloro-2-hydroxybenzyl)-3-ethyl-1-isopentyl-1,3,8-triazaspiro[4.5]decane-2,4-dione) and its 4,5 dichloro analogue, CWHM-505, are potent antimalarials (IC_50_ values against *Plasmodium falciparum* 3D7 of 0.310 μM and 0.099 μM) and the former features equivalent potency on the chloroquine-resistant Dd2 strain. In this study, the 1-benzylpiperidine moiety was replaced with naphthalene or a bicyclic indene system. Next, Molyneaux et al. [22] tested a broader range of substituted and unsubstituted aryl- and heteroaryl-piperazines. The results showed that 4-aminoquinoline-based heteroaryl-piperazines, in which the terminal secondary amino group is also unsubstituted, were found to be equally active against the chloroquine-resistant and chloroquine-sensitive strains. In this study 2,3-dihydro-1*H*-inden or benzo[*d*]imidazole was substituted at the piperazine moiety Mendoza et al. [23] evaluated piperazine and pyrrolidine derivatives (1-aryl-ketone, 1-aryl-alcohol and 1-aryl-oxime) for inhibition of the growth of the *Plasmodium falciparum* chloroquine-resistant (FCR-3) strain in culture. In this group of compounds, the combined presence of a hydroxyl group, a propane chain and a fluor appeared to be crucial for the antiplasmodial activity of the compounds. Shortening the carbon chain leads to a large decrease in activity. In this study, the carbon chain connected the hydantoin core with piperazine ring was elongated to a five-carbon chain.

We have been particularly interested in determining the influence of the designed modifications of hydantoin scaffold on physicochemical properties, antimalarial activity and toxicity against mammalian cells.

## 2. Materials and Methods

### 2.1. Chemicals and Reagents

DMSO and MTT (3-[4,5-dimethylthiazol-2-yl]-2,5-diphenyltetrazolium bromide) were purchased from Sigma-Aldrich (Poznań, Poland). The HaCaT cell line was obtained from AddexBio (San Diego, CA, USA). DMEM medium and FBS were from Biowest (Zgierz, Poland). GlutaMAX™ and 100× antibiotic-antimycotic were purchased from Life Technologies (Gibco/Life Technologies, Warsaw, Poland). All chemicals and solvents used for synthesis were purchased from commercial suppliers, namely, Sigma-Aldrich (Poznań, Poland) and Chempur (Piekary Śląskie, Poland) and were used without further purification.

### 2.2. General Experimental Procedures

Thin-layer chromatography (TLC) was performed on Merck silica gel 60 F_254_ aluminum sheets (Merck; Darmstadt, Germany), using the following mixtures of solvents: (S1) methylene chloride/methanol (9:0.7), (S2) methylene chloride/methanol (9:1.2). Analytical HPLC was conducted on a Waters HPLC instrument with a Waters 485 Tunable Absorbance Detector UV, equipped with a Symmetry column (C18, 3.5 µm, 4.6 mm × 30 mm) using a water/acetonitrile gradient with 0.1% TFA as the mobile phase, at a flow rate of 5 mL/min. The liquid chromatography/mass spectrometry (LC/MS) analysis was performed on a Waters Acquity TQD system, with a Waters TQD quadrupole mass spectrometer, with detection by UV (DAD) using an Acquity UPLC BEH C18 column (1.7 μm, 2.1 mm × 100 mm). A water/acetonitrile gradient with 0.1% TFA was used as the mobile phase, at a flow rate of 0.3 mL/min. NMR spectra were recorded on an FT-NMR 500 MHz spectrometer (Joel Ltd., Akishima, Tokyo, Japan); chemical shifts are expressed in parts per million (ppm), using the CDCl_3_ signal as an internal standard. The *J* values are expressed in Hertz. Signal multiplets are represented by the following abbreviations: s (singlet), brs (broad singlet), d (doublet), dd (doublet of doublets), dt (doublet of triplets), t (triplet), q (quintet), m (multiplet) (Supplementary Materials).

Arylpiperazine derivatives (Ia and Ib): 2-(piperazin-1-yl)-1H-benzo[d]imidazole, 1-(2,3-dihydro-1*H*-inden-2-yl)piperazine, and their detailed analytical data were described previously [24,25].

#### 2.2.1. General Procedure for Obtaining Spirohydantoin Derivatives (**1**, **2**)

General procedure for obtaining starting spirohydantoin compounds: (*R*,*S*)-2′,3′-dihydrospiro[imidazolidine-4,1′-indene]-2,5-dione (**1**), (*R*,*S*)-1′,3′-dihydrospiro[imidazolidine-4,2′-indene]-2,5-dione (**2**), and their detailed analytical data were described previously [14].

#### 2.2.2. General Procedure for Alkylation of Spirohydantoin (**3**, **4**)

Intermediate products (3, 4) were obtained in line with previously described N3-alkylate spirohydantoins, with slight modifications. Briefly, a mixture of the appropriate spirohydantoin (1, 2; 2.5 mmol) and potassium carbonate (0.69 g, 5 mmol) in acetonitrile (15 mL) was heated to 80 °C with stirring on a magnetic stirrer. After 30 min heating, an alkylating agent, 1.5-dibromopentane (0.632 g, 2.7 mmol), was added dropwise to the reaction mixture, which was kept at 80 °C for 8 h. Then, the reaction mixture was filtered, and the resulting filtrate was concentrated under vacuum. The resulting intermediate racemic compounds were purified by column chromatography on silica gel, using (S1) methylene chloride/methanol (9:0.7) as eluent.

- (*R*,*S*)-1-(5-bromopentyl)-3′,4′-dihydro-2′H-spiro[imidazolidine-4,1′-naphthalene]-2,5-dione (**3**)

Creamy powder. Yield: 72%; TLC: *R_f_* = 0.76 (S1); HPLC: *t_R_* = 1.613; ESI–S [M + H]^+^ calcd for 365.08, found: 365.12, 367.12; ^1^H NMR (500 MHz, CDCL_3_-*d*) δ ppm 1.43–1.56 (m, 2 H) 1.70 (dt, *J* = 14.68, 7.41 Hz, 2 H) 1.79–2.03 (m, 4 H) 2.21–2.38 (m, 2 H) 2.87 (q, *J* = 5.90 Hz, 2 H) 3.40 (t, *J* = 6.67 Hz, 2 H) 3.57 (t, *J* = 7.18 Hz, 2 H) 5.90 (s, 1 H) 7.04 (dd, *J* = 7.69, 1.54 Hz, 1 H) 7.12–7.25 (m, 3 H).

- (*R*,*S*)-1-(5-bromopentyl)-2′,3′-dihydrospiro[imidazolidine-4,1′-indene]-2,5-dione (**4**)

Creamy powder. Yield: 57%; TLC: *R_f_* = 0.73 (S1); HPLC: *t_R_* = 1.529; ESI-MS [M + H]^+^ calcd for 351.06, found: 351.16, 353.16; ^1^H NMR (500 MHz, CDCL_3_-*d*) δ ppm 1.40–1.53 (m, 2 H) 1.63–1.72 (m, 2 H) 1.83–1.94 (m, 2 H) 2.19–2.31 (m, 1 H) 2.65–2.76 (m, 1 H) 2.99–3.11 (m, 1 H) 3.18–3.32 (m, 1 H) 3.39 (t, *J* = 6.67 Hz, 2 H) 3.55 (t, *J* = 7.05 Hz, 2 H) 5.89 (s, 1 H) 7.12 (d, *J* = 7.44 Hz, 1 H) 7.20–7.27 (m, 1 H) 7.28–7.33 (m, 2 H).

#### 2.2.3. General Procedure for Obtaining the Final Compound Series (**5**–**8**)

The appropriate bromoalkyl derivatives of spirohydantoin (0.3 mmol), and triethylamine (0.061 g, 0.6 mmol) were dissolved in 4 mL of acetonitrile and arylpiperazine (0.33 mmol) was added. The reaction mixture was heated to 100 °C in a microwave synthesizer (CEM corporation, 200 W). After 1 h of heating, the reaction mixture was concentrated under vacuum. The final, racemic compounds obtained were further purified by column chromatography using the following eluent systems: (S1) methylene chloride/methanol (9:0.7), and (S2) methylene chloride/methanol (9:1.2).

- (*R*,*S*)-1-(5-(4-(1H-Benzo[d]imidazol-2-yl)piperazin-1-yl)pentyl)-3′,4′-dihydro-2′H-spiro[imidazolidine-4,1′-naphthalene]-2,5-dione (**5**)

Light-yellow solid. Yield: 71%; TLC: *R_f_* = 0.29 (S2); HPLC: *t*_R_ = 0.948; ESI-MS [M + H]^+^ calcd for 487.27, found: 487.15; ^1^H NMR (500 MHz, CDCl_3_-*d*) δ ppm 1.29–1.34 (m, 2 H) 1.45–1.52 (m, 2 H) 1.63–1.70 (m, 2 H) 1.73–1.81 (m, 1 H) 1.90–1.98 (m, 1 H) 2.18–2.26 (m, 2 H) 2.28–2.32 (m, 2 H) 2.46 (br. s., 4 H) 2.75–2.88 (m, 2 H) 3.51–3.56 (m, 6 H) 6.29 (br. s., 1 H) 6.97–7.04 (m, 4 H) 7.08–7.16 (m, 3 H) 7.16–7.20 (m, 1 H). ^13^C NMR (126 MHz, CDCl_3_-*d*) δ ppm 19.13 (s) 24.53 (s) 26.07 (s) 27.98 (s) 28.86 (s) 34.28 (s) 38.60 (s) 46.55 (s) 52.34 (s) 58.29 (s) 62.78 (s) 112.50 (s.) 121.14 (s) 126.43 (s) 126.95 (s) 128.78 (s) 129.91 (s) 133.07 (s) 138.24 (s) 155.39 (s) 156.92 (s) 176.43 (s).

- (*R*,*S*)-1-(5-(4-(1H-Benzo[d]imidazol-2-yl)piperazin-1-yl)pentyl)-2′,3′-dihydrospiro[imidazolidine-4,1′-indene]-2,5-dione (**6**)

Light-yellow solid. Yield: 65%; TLC: *R_f_* = 0.27 (S2); HPLC: *t*_R_ = 0.928; ESI-MS [M + H]^+^ calcd for 473.26, found: 473.19; ^1^H NMR (500 MHz, CDCl_3_-*d*) δ ppm 1.29–1.37 (m, 2 H) 1.48–1.56 (m, 2 H) 1.63–1.71 (m, 2 H) 2.31–2.37 (m, 2 H) 2.51 (d, *J* = 3.44 Hz, 4 H) 3.03 (d, *J* = 16.32 Hz, 2 H) 3.50–3.64 (m, 8 H) 5.87–5.98 (m, 1 H) 7.02–7.06 (m, 2 H) 7.20 (s, 4 H) 7.30 (dd, *J* = 5.73, 3.15 Hz, 2 H). ^13^C NMR (126 MHz, CDCl_3_-*d*) δ ppm 24.46 (s) 25.85 (s) 27.90 (s) 30.15 (s) 36.82 (s) 38.39 (s) 45.90 (s) 52.31 (s) 58.38 (s) 71.23 (s) 112.29 (s) 120.76 (s) 122.54 (s) 125.43 (s) 127.29 (s) 129.52 (s) 140.07 (s) 144.17 (s) 155.81 (s) 157.26 (s) 176.33 (s).

- (*R*,*S*)-1-(5-(4-(2,3-Dihydro-1H-inden-2-yl)piperazin-1-yl)pentyl)-3′,4′-dihydro-2′H-spiro[imidazolidine-4,1′-naphthalene]-2,5-dione (**7**)

Light-yellow solid. Yield: 61%; TLC: *R_f_* = 0.24 (S1); HPLC: *t*_R_ = 1.002; ESI-MS [M + H]^+^ calcd for 487.29, found: 487.29; ^1^H NMR (500 MHz, CDCl_3_-*d*) δ ppm 1.23–1.40 (m, 4 H) 1.57 (dt, *J* = 15.25, 7.70 Hz, 2 H) 1.69 (q, *J* = 7.52 Hz, 2 H) 1.76–1.83 (m, 1 H) 1.93–2.00 (m, 1 H) 2.22–2.29 (m, 2 H) 2.35–2.40 (m, 2 H) 2.60 (br. s, 6 H) 2.83–2.93 (m, 4 H) 3.03–3.09 (m, 2 H) 3.19 (q, *J* = 8.09 Hz, 1 H) 3.56 (t, *J* = 7.16 Hz, 2 H) 5.71 (br. s, 1 H) 7.03 (d, *J* = 7.16 Hz, 1 H) 7.10–7.13 (m, 2 H) 7.14–7.18 (m, 4 H) 7.20–7.24 (m, 1 H). ^13^C NMR (126 MHz, CDCl_3_-*d*) δ ppm 19.17 (s) 24.72 (s) 26.12 (s) 28.08 (s) 28.89 (s) 34.31 (s) 37.03 (s) 38.67 (s) 51.18 (s) 52.93 (s) 58.38 (s) 62.72 (s, 5 C) 66.98 (s) 124.47 (s, 6 C) 126.54 (s) 127.00 (s) 128.78 (s) 129.89 (s) 133.11 (s) 138.22 (s) 141.49 (s) 156.86 (s) 176.26 (s).

- (*R*,*S*)-1-(5-(4-(2,3-Dihydro-1H-inden-2-yl)piperazin-1-yl)pentyl)-2′,3′-dihydrospiro[imidazolidine-4,1′-indene]-2,5-dione (**8**)

Light-yellow solid. Yield: 59%; TLC: *R_f_* = 0.24 (S1); HPLC: *t_R_* = 0.937; ESI-MS [M + H]^+^ calcd for 473.26, found: 473.19; ^1^H NMR (500 MHz, CDCl_3_-*d*) δ ppm 1.32 (quin, *J* = 7.73 Hz, 2 H) 1.52 (quin, *J* = 7.66 Hz, 2 H) 1.66 (quin, *J* = 7.52 Hz, 2 H) 2.19–2.26 (m, 1 H) 2.30–2.35 (m, 2 H) 2.36–2.77 (m, 8 H) 2.87 (dd, J = 15.04, 8.74 Hz, 2 H) 2.98–3.08 (m, 4 H) 3.12–3.19 (m, 1 H) 3.24 (dt, *J* = 15.97, 8.20 Hz, 1 H) 3.52 (t, *J* = 7.30 Hz, 2 H) 5.93 (s, 1 H) 7.08–7.13 (m, 3 H) 7.13–7.18 (m, 2 H) 7.20–7.24 (m, 1 H) 7.28–7.32 (m, 2 H). ^13^C NMR (126 MHz, CDCl_3_-d) δ ppm 24.55 (s) 25.49 (s) 27.97 (s) 36.82 (s) 38.61 (s) 44.44 (s) 50.42 (s) 52.47 (s) 58.07 (s) 66.72 (s) 68.43 (s) 124.49 (s) 124.73 (s) 126.00–127.91 (m) 127.15–128.67 (m) 139.00 (s) 141.14 (s) 156.39 (s) 176.01 (s).

### 2.3. pK_a_ and Log D Calculation

The negative logarithm of the acid dissociation constant (p*K*_a_) and the decimal logarithm distribution coefficient (log *D*) were calculated using the MarvinSketch software (version 20.12.0, ChemAxon Ltd., Cambridge, MA, USA).

### 2.4. P. falciparum Cultures and Drug Susceptibility Assay

*Plasmodium falciparum* cultures were carried out according to Trager and Jensen, with slight modifications [26]. The CQ-susceptible strain D10 and the CQ-resistant strain W2 were maintained at 5% hematocrit (human type A-positive red blood cells) in RPMI 1640 (EuroClone, Milan, Italy) medium with the addition of 1% AlbuMax (Invitrogen, Milan, Italy), 0.01% hypoxanthine, 20 mM Hepes, and 2 mM glutamine. All the cultures were maintained at 37 °C in a standard gas mixture consisting of 1% O_2_, 5% CO_2_, and 94% N_2_. Compounds were dissolved in DMSO and then diluted with medium to achieve the required concentrations (final DMSO concentration < 1%, which is non-toxic to the parasite). Drugs were placed in 96-well flat-bottomed microplates starting from the concentration of 20 µg/mL and 8 serial two-fold dilutions were made. Asynchronous cultures with parasitaemia of 1–1.5% and 1% final hematocrit were aliquoted into the plates and incubated for 72 h at 37 °C. Parasite growth was determined spectrophotometrically (OD_650_) by measuring the activity of the parasite lactate dehydrogenase (pLDH), according to a modified version of the method of Makler in control and drug-treated cultures [27]. The antimalarial activity is expressed as 50% inhibitory concentrations (IC_50_); each IC_50_ value is the mean of at least three separate experiments performed in duplicate.

### 2.5. Cytotoxicity Assay

HaCaT cells were cultured in DMEM medium with 10% FBS, GlutaMAX™ and antibiotic-antimycotic. Cells were cultured at 37 °C in a humidified atmosphere containing 5% CO_2_. To evaluate the cytotoxic activity of tested compounds, the MTT assay was used [28]. Cells (5 × 10^3^ cells/well) were plated into a 96-multiwell plate and incubated for 24 h, to grow as monolayers. Subsequently, different doses of compounds dissolved in DMSO were added to each well and mixed. Plates were then incubated for 48 h. The culture media with tested samples were then removed, and cells in each well were treated with 50 μL of complete culture medium with MTT. After 4 h of incubation in a cell culture incubator, MTT solution was replaced with DMSO (50 μL/well) and plates were agitated for 5 min to dissolve the formazan salts. Formazan absorbance was measured at 570 nm, with background measured at 630 nm, using an Asys UVM 340 Microplate Reader (Cambridge, UK). Results were expressed as IC_50_ values, with respect to the control (not treated cells).

### 2.6. Hemolysis Assay

The study protocol was approved by the Bioethics Commission at the Lower Silesian Medical Chamber (1/PNHAB/2018). Hemolytic activity was estimated by the measurement of hemoglobin release from human erythrocyte suspensions after incubation with (5), (6), (7) and (8), as previously described [29]. Compouds dissolved in DMSO were added in a volume corresponding to a final concentration of 20 µg/mL in the sample (DMSO was added in an identical volume). Negative and positive controls were also determined. Hemolysis (H) was calculated according to the following formula:

H (%) = [(A_sample_ − A_negative control_)/(A_positive control_ − A_negative control_)] × 100 (1)

where A_sample_, A_negative control_, and A_positive control_ represent the absorbances of the sample, and negative (erythrocytes in PBS buffer) and positive (erythrocytes in distilled water) controls, respectively.

## 3. Results and Discussion

### 3.1. Chemistry

**Scheme 1:**
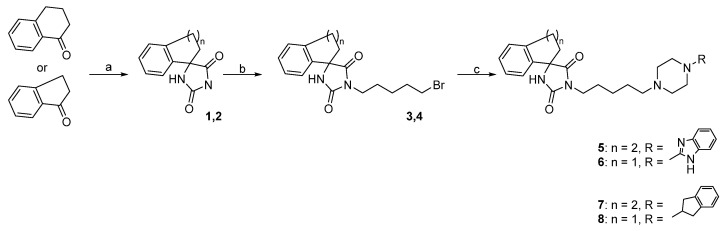
Reagents and conditions for compounds 8–15: (**a**) KCN, (NH_4_)_2_CO_3_, 50% EtOH, 56–60 °C, 28 h; (**b**) 1,5-dibromopentane, K_2_CO_3_, MeCN, 80 °C, 8 h; (**c**) arylpiperazine, TEA, MeCN, 100 °C, 1 h, MW.

Synthetic routes and chemical structures of the designed spirohydantoin derivatives (**5**–**8**) are depicted in Scheme 1. Spirohydantoin rings (**1** and **2**) were synthesized from commercially available ketones, in a Bucherer-Berg reaction, using the modification of Goodson et al. Then, spirohydantoins (**1**, **2**) were coupled with 1,5-dibromopenane, to give alkylated intermediate compounds **3** and **4**. Finally, racemic compounds (**5**–**8**) were obtained through coupling intermediates (3 or 4) with corresponding arylpiperazines, namely, 2-(piperazin-1-yl)-1H-benzo[d]imidazole or 1-(2,3-dihydro-1*H*-inden-2-yl)piperazine, whose analytical data were in line with previously described procedures [24,25].

The applied synthetic methods facilitated the synthesis of intermediates (**3**, **4**) and finally, compounds (**5**–**8**), with an average-to-good yield (57–71% and 59–72%, respectively). The purity of the final compounds (**5**–**8**) was above 95%.

Designing compounds with well-balanced physicochemical parameters is crucial for biologically active compounds, because these parameters determine their absorption, distribution, metabolism and excretion [30]. One of the key physicochemical parameters is lipophilicity, expressed as the partition coefficient (Log *P*), or distribution coefficient (Log *D*). The log *P* parameter relates to non-ionized molecules and the log *D* takes into account ionized and non-ionized particles at a given pH. Thus, log *D* is a better descriptor than log *P*, which reflects the partitioning of a mixture of drug species as well as the actual drug lipophilicity at any given pH.

For compounds (**5**–**8**), as well as for CQ, the log *D* values at representative pH values for blood (7.4), cytoplasm (7.2), and *P. falciparum* food vacuole (FV) (5.5) were calculated (Table 2). FVs degrade host haemoglobin via a series of peptidases during the erythrocytic life stages of the parasite. Thus, we wished to establish the role of a possible accumulation of compounds (**5**–**8**) in the FV. It is known that CQ accumulates in the FV, however only a neutral form of CQ can diffuse across the FV membrane, where, in the acidic environment of the FV, it is protonated and is subsequently unable to freely diffuse out of the FV again. Thus, according to the log *D* values for compounds (**5**–**8**) under pH conditions representative of blood, cytoplasm and FV, it can be concluded that, like CQ, the compounds would favor accumulation in the FV of the parasite. Based on the data in Table 1 and Table 2, it can be concluded that compounds (**5**–**8**), as well as Q and CQ, display a greater affinity for a lipophilic environment, with the Log *D* values of the final compounds being 3-fold higher than that of the reference drugs. However, Log *D* values of compounds (**5**–**8**) are similar to the Log *D* value of WR182393, which is quite an active antimalarial compound.

Another important physicochemical parameter is p*K*_a_, which describes the ionization state of a compound at a specific pH. At blood pH, the acidic groups of the final compounds (**5**–**8**) will be non-ionized, while their amino groups will be partially ionized. Such a situation, on the one hand, will improve the solubility of compounds (**5**–**8**) in the blood, but on the other hand, it may hinder their penetration through biological membranes.

### 3.2. Biological Activities

The chloroquine sensitive (D10) and chloroquine resistant (W2) strains of *P. falciparum* were used to establish the potential activity of the studied compounds against blood-stage parasites in vitro. The IC_50_ values for compounds (**5**–**8**) were subsequently calculated, as well as their respective RIs (resistance indexes) between the sensitive and resistant strain, respectively, as summarized in Table 3, whereas representative dose response activities of compounds (**5**), (**6**) or (**7**) are included in the Supplementary Materials. The determined IC_50_ values against the D10 strain were in the range of 6202.00–9659.70 ng/mL, whereas the values obtained for the W2 strain were in the range of 2424.15–5648.07 ng/mL. Interestingly, the highest inhibition of both strains of *P. falciparum* was observed for the same compound, namely (**5**). Importantly, the estimated IC_50_ value for W2 was similar to the value obtained for WR 182393 (2664.69 ng/mL) to the same strain, as well [31]. Another extremely important finding is that the tested agents are more active against a *Plasmodium* strain resistant to CQ. This is directly reflected in their low RI values (0.39–0.58) while, for CQ, this value is 13.44. Moreover, structure-activity relationships for the designed compounds revealed that the presence of naphthalene and benzo[d]imidazole-2-yl-piperazine moieties were necessary for the most promising antiplasmodial activities.

One of the greatest public health problems in the control of malaria is the the spread of resistance to antimalarial drugs used on a large scale [32]. Therefore, our results are very promising, especially in view of the fact that chloroquine resistant *P. falciparum* strains have been described almost everywhere.

As the studied agents have shown encouraging antiplasmodial activities, we further assessed their cytotoxic effects using the MTT assay. Additionally, for each compound, the selectivity index (SI), namely the ratio between the IC_50_ values on the human cells and that on the *P. falciparum* strains, was calculated and reported in Table 3. For the cytotoxicity studies, we choose the HaCaT keratinocyte cell line, since skin cell lines are often used as a control human cell line for antimalarial agents [33,34]. The cytotoxicity test showed 50% inhibitory cellular cytotoxicity at a concentration range of 20,216.67 to 30,536.67 ng/mL, after 48 h. The calculated selectivity indexes for D10 were similar, in the range of 3.16–3.32 while, in the case of W2, they reached much higher values (4.02–8.34), which suggests they demonstrate significant selectivity towards W2, especially (**5**), in relation to the CQ-resistant strain. Again, (**5**) had the highest selectivity index which, together with its low toxicity, makes this agent the most promising compound amongst the studied series.

The synthesized compounds were subsequently evaluated for their effects on human erythrocytes. In order to estimate this, fresh erythrocytes were treated with compounds (**5**–**8**) for 30 min, at a concentration of 20 µg/mL. It was shown that compound (**5**) caused minimal hemolysis, on the level of 5.6%, at a concentration corresponding to 3.2-fold (D10) and 8.2-fold (W2) of the IC_50_ of the respective *Plasmodium* strains. The other derivatives (**6**–**8**) had a hemolysis level of less than 2.7% at the same concentration. In summary, we can conclude that compounds (**5**–**8**) are not toxic to human erythrocytes at a concentration at which they effectively inhibit the growth of *P. falciparum*. This is a very valuable observation, especially considering that some artemisinin derivatives can cause hemolysis [35]. Moreover, we can exclude that the observed antimalarial effect of the tested compounds was due to erythrocyte hemolysis.

In this work, we synthesized a series of bioinspired hydantoin derivatives and further studied their antimalarial activities, as well as their cytotoxicity and hemolytic effects. Compound (**5**) turned out to be the best hit compound in the series, due to the highest activity against *P. falciparum* strains, especially the CQ-resistant strain, and low toxicity to mammalian cells. Due to its low solubility in water, further work will be carried out to develop an appropriate formulation that will improve its bioactivity and bioavailability.

## Figures and Tables

**Table 1:** Antimalarial drugs based on a 4-aminoquinoline scaffold and compounds from references [2,3,4,5,6,7,8,9,10,11]. Chemical structures and physicochemical properties.

Compound	Structure	MW	p*K*_a_	Log *D* pH 7.4
(Q) Quinine	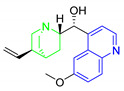	324.41	13.89	0.86
			9.05	
			4.02	
(CQ) Chloroquine	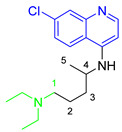	319.87	10.32	0.88
			7.29	
(PQ) Primaquine	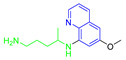	259.35	10.20	−1.13
			4.09	
			0.60	
(AQ) Amodiaquine	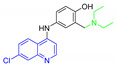	355.86	10.17	2.32
			9.10	
			6.46	
(MQ) Mefloquine	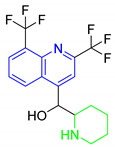	378.31	13.79	2.07
			9.46	
WR182393	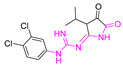	341.19	12.69	3.12
			4.37	
General structure of imidazolidin-4-ones	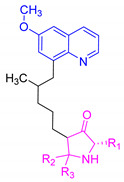	-	-	-
TDR32750	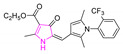	418.41	No ionizable atoms found	3.97
Aplysinopsin	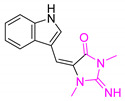	254.29	15.84	1.74
			2.93	

**Table 2:** The structures and physicochemical properties of the final imidazolidinedione derivatives (**5**–**8**).

Compound	Structure	p*K*a	Log D		
			pH 7.4	pH 7.2	pH 5.5
(**5**)	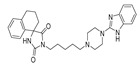	12.32	3.39	3.20	1.11
		11.11			
		8.41			
		5.86			
(**6**)	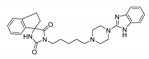	12.32	2.99	2.80	0.72
		11.03			
		8.41			
		5.86			
(**7**)	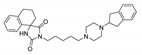	11.14	3.00	2.81	1.20
		8.64			
		2.86			
(**8**)	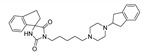	11.05	2.60	2.41	0.80
		8.64			
		2.86			
CQ		-	0.88	0.64	−0.76

**Table 3:** In vitro antimalarial activity of compounds (**5**–**8**) against the D10 (CQ-sensitive) and W2 (CQ-resistant) strains of *P. falciparum*, cytotoxicity on HaCaT cells and relevant RI and SI.

Compound	*P. falciparum* IC_50_ (ng/mL)		RI ^a^	HaCaT IC_50_ (ng/mL)	SI ^b^	
	D10	W2			D10	W2
(**5**)	6202.00 ± 892.09	2424.15 ± 255.34	0.39	20,216.67 ± 1181.54	3.26	8.34
	12.75 ± 0.02 ^c^	4.98 ± 0.52 ^c^		41.55 ± 2.43 ^c^		
(**6**)	8269.85 ± 736.88	4782.00 ± 97.44	0.58	27,406.67 ± 701.17	3.31	5.73
	17.50 ± 1.55 ^c^	10.12 ± 0.20 ^c^		57.99 ± 1.48 ^c^		
(**7**)	9659.70 ± 140.86	4346.50 ± 659.31	0.45	30,536.67 ± 1033.89	3.16	7.02
	19.85 ± 0.29 ^c^	8.93 ± 1.35 ^c^		62.75 ± 2.12 ^c^		
(**8**)	>10,000	5648.07 ± 1946.84	-	22,723.33 ± 1175.00	-	4.02
		11.95 ± 4.12 ^c^		48.08 ± 2.49 ^c^		
CQ	7.64 ± 1.70	102.67 ± 27.49	13.44	*nd*	*nd*	*nd*
	0.01 ± 0.003 ^c^	0.20 ± 0.05 ^c^				

^a^ RI = IC_50_ CQ resistant *P. falciparum* strain/IC_50_ CQ sensitive *P. falciparum* strain, ^b^ SI = IC_50_ HaCaT/IC_50_ *P. falciparum* strain., ^c^ IC_50_ expressed in (µM).

## Data Availability

The data presented in this study are available in Supplementary Materials.

## Supplementary Materials

The following are available online at https://www.mdpi.com/2218-273X/11/1/33/s1. ^1^H- and ^13^C-NMR spectra for compounds **5**, **6**, **7** and **8** provided.
