# Supplementary material for: Synthesis and Antiplasmodial Activity of Novel Bioinspired Imidazolidinedione Derivatives
Source: Biomolecules. 2020 Dec 29;11(1):33. doi: 10.3390/biom11010033 (PMC7823712; doi:10.3390/biom11010033)
Supplement: Supplementary file 1 [file biomolecules-11-00033-s001.pdf]

Supplementary Materials

Synthesis and Antiplasmodial Activity of Novel Bioinspired Imidazolidinedione Derivatives

Anna Jaromin [1,*], Anna Czopek [2], Silvia Parapini [3], Nicoletta Basilico [4], Ernest Misiak [2], Jerzy Gubernator [1] and Agnieszka Zagórska [2]

[1] Department of Lipids and Liposomes, Faculty of Biotechnology, University of Wroclaw, 50-383 Wroclaw, Poland; anna.jaromin@uwr.edu.pl (A.J.), jerzy.gubernator@uwr.edu.pl (J.G.)
[2] Department of Medicinal Chemistry, Faculty of Pharmacy, Jagiellonian University Medical College, Medyczna 9 str, 30-688 Kraków, Poland anna.czopek@uj.edu.pl (A.C.), ernest.misiak@student.uj.edu.pl (E.M.), agnieszka.zagorska@uj.edu.pl (A.Z.)
[3] Dipartimento di Scienze Biomediche per la Salute, Università di Milano, Via Pascal 36, 20133 Milan, Italy; silvia.parapini@unimi.it
[4] Dipartimento di Scienze Biomediche, Chirurgiche e Odontoiatriche, Università di Milano, Via Pascal 36, 20133 Milan, Italy; nicoletta.basilico@unimi.it
* Correspondence: anna.jaromin@uwr.edu.pl; Tel.: +48-71-3756203

Herein, we presented proton and carbon nuclear magnetic resonance (^{1}H and ^{13}C NMR) spectra for compounds (5), (6), (7) and (8).

Compound 5

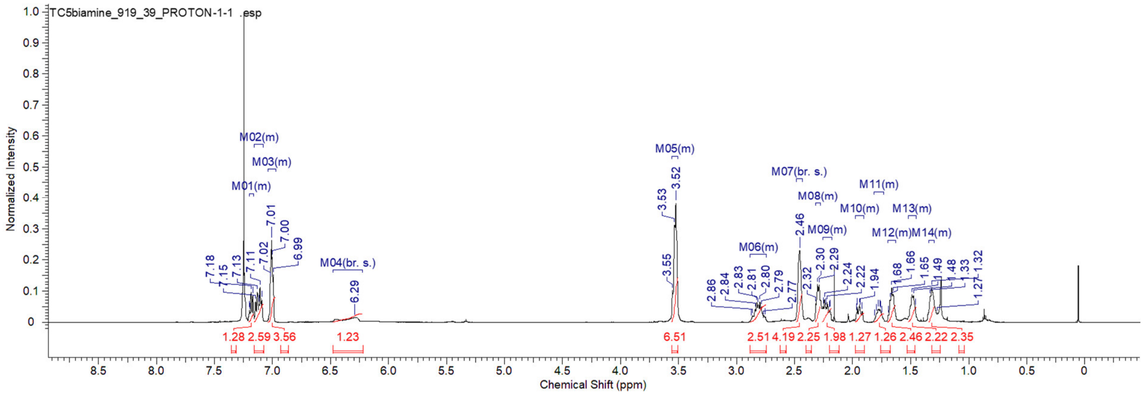

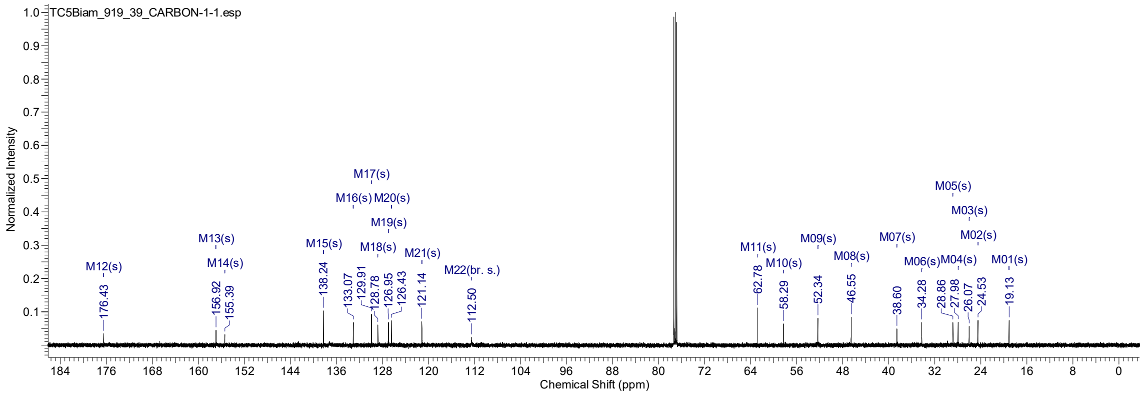

Compound 6

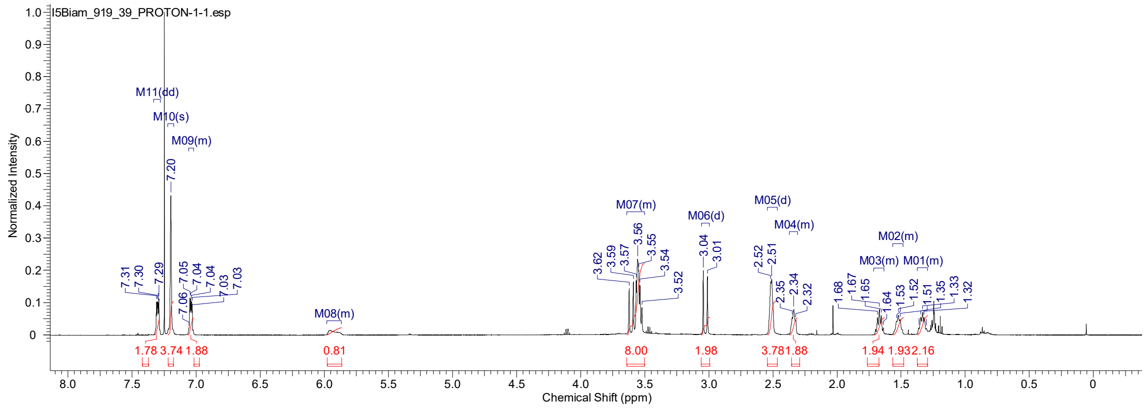

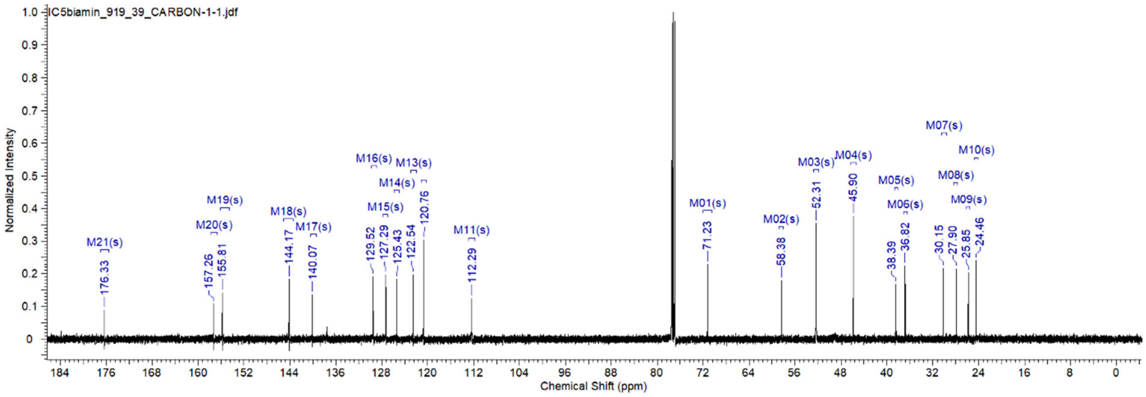

Compound 7

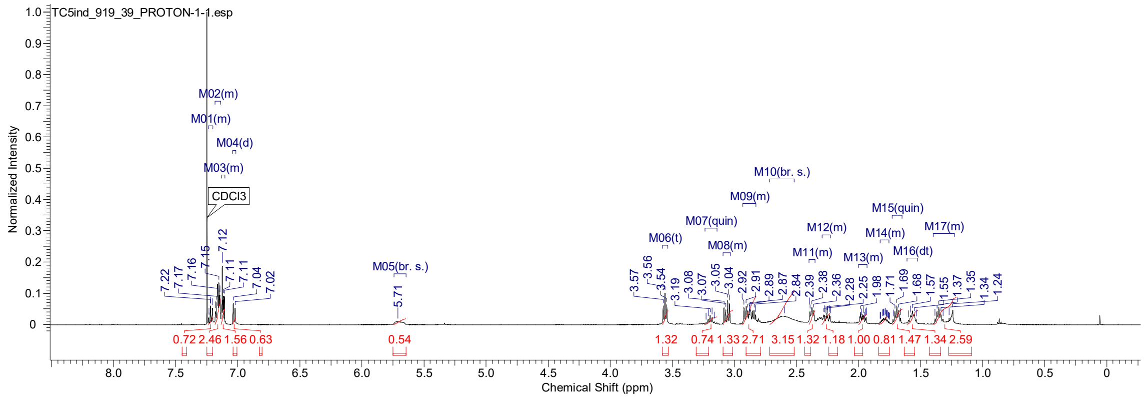

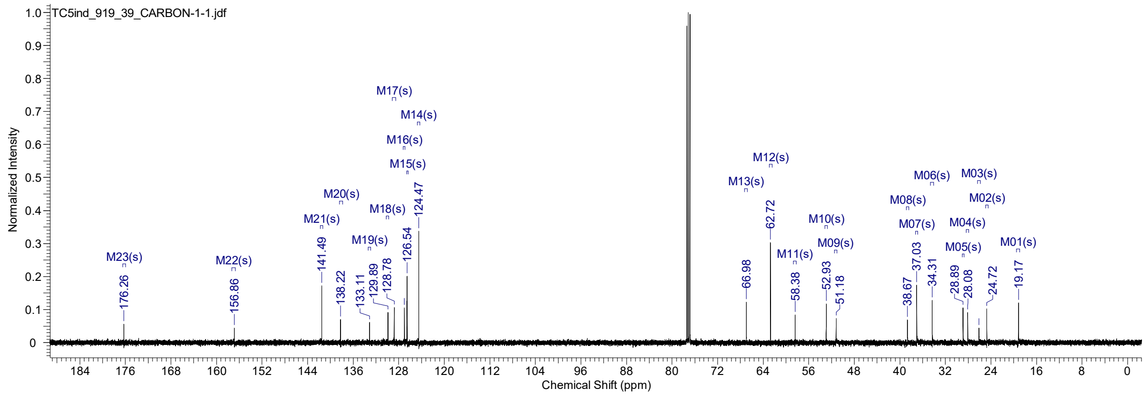
TC5ind_919_39_CARBON-1-1.jdf
Normalized Intensity
Chemical Shift (ppm)

Compound 8

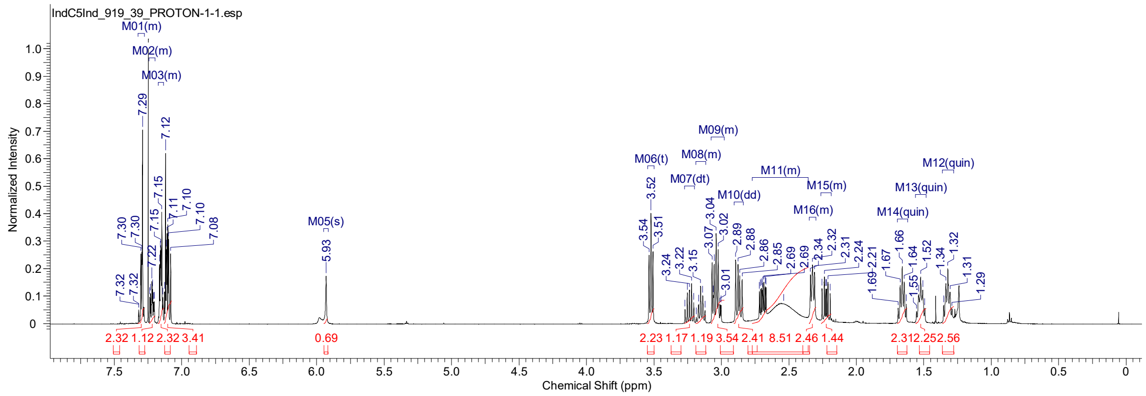
IndC5Ind_919_39_PROTON-1-1.esp
Normalized Intensity
Chemical Shift (ppm)

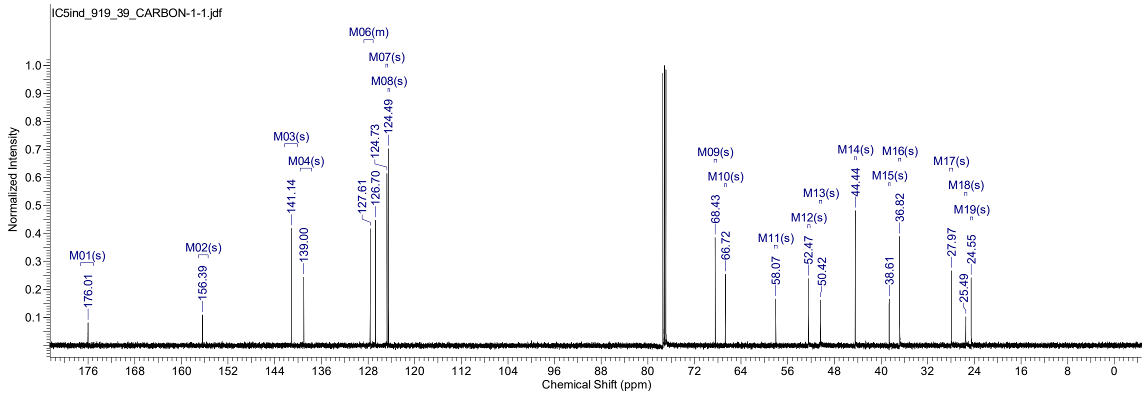
IC5ind_919_39_CARBON-1-1.jdf
Normalized Intensity
Chemical Shift (ppm)

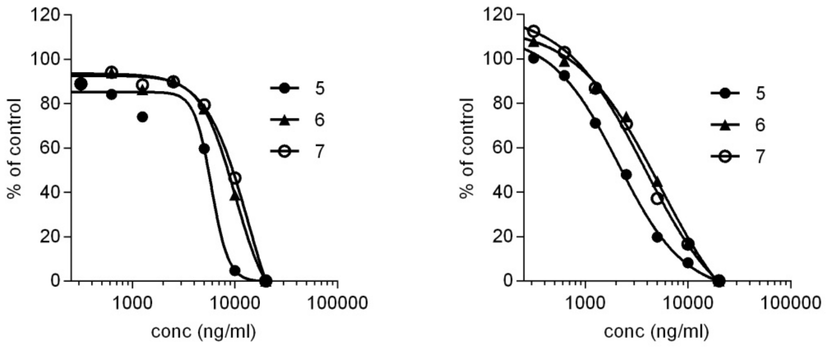

A **B**

Dose response activity of compounds (5), (6) and (7) against the *P. falciparum* chloroquine-sensitive strain D10 (panel A) and chloroquine-resistant strain W2 (panel B). The results are expressed as % of parasite growth with respect to the control, in the presence of increasing concentrations of the molecules. This is a representative experiment of at least three independent experiments each involving duplicate sample measurements.
